# Experimental Investigation of Polymer-Modified Bituminous Stone Mastic Asphalt Mixtures Containing Cellulose Acetate Recycled from Cigarette Butts

**DOI:** 10.3390/ma18235340

**Published:** 2025-11-27

**Authors:** Hande Varol Morova, Cengiz Özel

**Affiliations:** Department of Civil Engineering, Faculty of Technology, Isparta University of Applied Sciences, 32100 Isparta, Türkiye; cengizozel@isparta.edu.tr

**Keywords:** stone mastic asphalt, cigarette butt, Elvaloy ret, polyphosphoric acid, binder drain-down, moisture damage

## Abstract

Stone Mastic Asphalt (SMA) mixtures exhibit superior performance under traffic loads due to the high content of coarse aggregates; however, the high bitumen content also leads to the problem of bitumen drainage from the mixture. Several studies have been conducted on the use of stabilizing additives such as fibers, polymers, or mineral fillers to reduce binder drainage in SMA mixtures. In this study, however, an innovative and sustainable solution was developed to address the bitumen drainage problem encountered in SMA pavements and to improve the long-term performance of the mixture. In this context, the feasibility of using cellulose acetate (SG) material recycled from cigarette butts as an alternative fiber additive to the traditionally used cellulose fiber (SL) was investigated. This method aims to achieve both environmental benefits in terms of waste management and economic advantages in terms of additive materials. Additionally, the effect of using different SL contents (0.1%, 0.2%, 0.3%, 0.4%, 0.5%) on mixture performance was examined. Within this scope, both pure bitumen (B) and Elvaloy RET + PPA (E)-modified bitumen (1.6%, 1.7%, and 1.8% Elvaloy RET + 0.2% PPA) were used to produce both fiber-reinforced and non-fiber-reinforced SMA mixtures. Traditional and Superpave tests were conducted to determine the rheological and physical properties of the pure and modified binders. All SMA specimens were tested for Marshall stability and flow, Marshall quotient, indirect tensile strength (ITS), tensile strength ratio (TSR), Schellenberg bitumen drainage, sand patch, and Cantabro particle loss. Furthermore, a cost analysis was carried out to evaluate the economic effect of different fiber types and proportions. Among the SMA mixtures, the highest stability and resistance to moisture damage were achieved in the mixtures containing 1.6% Elvaloy RET + 0.2% PPA with 0.3–0.4% SG and 1.7% Elvaloy RET + 0.2% PPA with 0.3–0.4% SL, while the optimum surface texture depth was obtained in the mixtures containing 1.6–1.7% Elvaloy RET + 0.2% PPA with 0.3% SG. In conclusion, the Elvaloy RET + PPA modification enhanced the aging resistance of the bitumen, while the SG fibers used at 0.3–0.4% fiber content in the 1.6–1.7% Elvaloy RET + 0.2% PPA-modified series were identified as a promising mechanical and economic alternative to conventional SL fibers.

## 1. Introduction

The increasing traffic loads, climate change effects, and global sustainability requirements have led to the development of high-performance and environmentally friendly pavement materials. Among these, Stone Mastic Asphalt (SMA) mixtures have gained widespread acceptance since their introduction in Germany in the 1960s to mitigate rutting problems caused by studded tires [1]. Due to their high coarse aggregate and filler content, SMA mixtures exhibit superior durability and deformation resistance, particularly when modified bitumen is used. However, compared to conventional asphalt mixtures, SMA production requires higher costs due to the greater proportion of asphalt binder used in the mixture, as well as the effects of higher mixing temperatures, compaction difficulties, and the use of cellulose fibers to prevent drainage [2].

To improve the performance of bituminous mixtures, polymer modification is one of the most widely adopted techniques. Polymers enhance the viscoelastic behavior of bitumen, reduce temperature susceptibility, and improve aging and deformation resistance [3,4]. Şengöz and Işıkyakar (2008) reported that Styrene–Butadiene–Styrene (SBS) and Ethylene–Vinyl Acetate (EVA) polymers improved the softening point and Marshall stability of bitumen [5]. Similarly, Kordi and Shafabakhsh (2017) observed that Nano Fe_2_O_3_ increased the stiffness modulus and rutting resistance, while Arshad et al. (2019) found that nanosilica addition enhanced the elastic modulus [6,7]. Gong et al. (2018) reported that adding 0.5–3% Carbon Nanotube (CNT) decreases penetration, increases the softening point, and enhances high-temperature performance and aging resistance [8].

Recent research has focused on Elvaloy RET (Reactive Elastomeric Terpolymer) and Polyphosphoric Acid (PPA) as promising modifiers for bitumen. Irfan et al. (2017) identified 1% Elvaloy RET as the optimum dosage, providing the best rutting and temperature resistance [9]. Geçkil (2019) found that 0.4–1.2% Elvaloy RET improved elasticity and high-temperature performance [10]. Taciroğlu (2023) reported that 1.6–1.8% Elvaloy RET combined with 4–5% SBS enhanced both high-temperature and aging resistance, while Vachhani and Mishra (2014) found that 2% Elvaloy RET achieved the best stiffness and elasticity [11,12]. Inocente Domingos and Faxina (2015) and Jasso et al. (2015) also confirmed that Elvaloy RET + PPA modification improved the rheological properties and high-temperature deformation resistance of bitumen [13,14].

Numerous studies have been conducted to address the problem of binder drain-down in SMA, and researchers have reached a consensus on the use of polymer-modified bitumen or fibers [15]. Fauzi et al. (2020) reported that 0.2% cellulose fiber minimized abrasion loss, while 0.3% provided the highest modulus [16]. Gallo et al. (2021) found that incorporating jute fiber within the 0.1–0.3% range improved the mixture’s durability, and the best stiffness modulus performance was obtained with the mixture containing 0.2% fiber; Shaffie et al. (2021) concluded that 0.3% steel fibers improved stiffness and stability; Ma et al. (2023) observed that a hybrid mixture containing 0.3% basalt and 0.2% cellulose fibers yielded the lowest deformation in the Hamburg wheel-tracking test [17,18,19]. Similarly, Alonso-Troyano et al. (2025) found that incorporating 0.3% recycled cotton fibers significantly reduced binder drainage compared to mixtures without fibers, while also demonstrating high resistance to moisture damage (indirect tensile strength ratio = 96.3%), making it suitable for temperate and humid climates [20].

**Figure 1:**
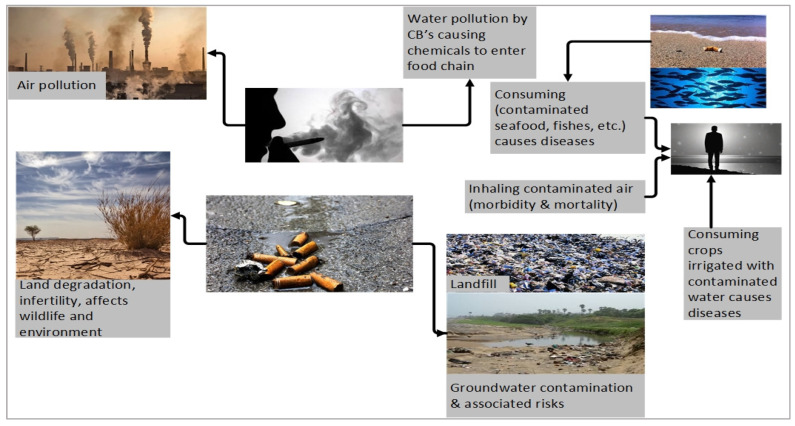
Environmental impacts of cigarette waste [24].

In the pursuit of sustainable pavement technologies, the reuse of waste materials in asphalt mixtures has become an important research direction. Zoorob and Suparma (2000) investigated asphalt concrete mixtures incorporating recycled low-density polyethylene (LDPE) as a partial aggregate replacement and reported that a 30% volumetric substitution of LDPE reduced the density by 16% and increased the Marshall stability by 250%, thereby enhancing deformation resistance [21]. Morova (2024) evaluated waste phonolite (PW) as a filler material in hot mix asphalt and found that it can be effectively used as a sustainable filler for pavements subjected to low traffic loads [22]. Rodríguez et al. (2024) utilized residual cotton fibers obtained from the Peruvian textile industry as an alternative to commercial fibers in SMA mixtures and demonstrated that a 0.20% fiber content provided optimal performance, improving tensile strength and rutting resistance [23]. Among these waste materials, cigarette butts (CBs) represent one of the most problematic urban wastes, with approximately 4.5 trillion discarded annually, causing significant environmental pollution due to their slow degradation and toxic leachate. The environmental impacts of cigarette butts are illustrated in Figure 1 [24]. The filters are mainly composed of cellulose acetate, a semi-synthetic polymer derived from natural cellulose, which can persist for years in soil and water [25].

Recent studies have explored the use of cigarette-butt-derived cellulose acetate fibers as a recycled additive in asphalt mixtures. Rahman et al. (2020) and Mohajerani et al. (2022) found that these fibers are compatible with bitumen and improve mixture stability [26,27]. Gallo and Valentin (2024) reported enhanced mechanical performance, while Jin et al. (2019) observed higher stiffness and durability [28,29]. Tataranni and Sangiorgi (2021) also demonstrated the potential use of recycled cigarette butts in hot mix asphalt [30]. Guo et al. (2024) found that 15 mm non-plastic electronic cigarette butts (E-CB) fibers achieved the highest indirect tensile strength and stiffness, Al-Ameri and Ramadhansyah (2022) reported optimum performance at 5% CB addition, and Hu et al. (2025) concluded that 1–4% CB fibers improved rheological properties, though slightly reduced storage stability [31,32,33].

In this context, the present study proposes an innovative and sustainable approach by using recycled cellulose acetate (SG) fibers obtained from cigarette butts as an alternative to traditional cellulose (SL) fibers in SMA mixtures. The primary objective is to reduce binder drain-down while promoting the recycling of environmentally harmful waste materials. Both pure and Elvaloy RET + PPA-modified bitumen were used, with fiber contents ranging from 0.1% to 0.5% and polymer dosages of 1.6–1.8%. A comprehensive experimental program was conducted, including density, air voids, Marshall stability, moisture damage, Schellenberg drain-down, sand patch texture, and Cantabro abrasion tests.

## 2. Materials and Methods

### 2.1. Aggregate

In this study, basalt aggregate was used as coarse and fine aggregate, while limestone dust was employed as filler. Both the limestone and basalt aggregates used in the study were supplied by the Municipality of Isparta. The physical and mechanical properties of the aggregates were determined in accordance with the relevant ASTM and TS EN standards (Table 1). The aggregate gradation was prepared in accordance with the limits specified by the Turkish Highway Technical Specification (HTS) [34] and is shown in Table 2.

### 2.2. Bitumen

In the production of SMA, the unmodified bitumen must comply with the TS EN 12591 standard [44] and belong to the 40–60 or 50–70 penetration grade. The polymer-modified bitumen, on the other hand, must meet the requirements of the TS EN 14023 standard [45]. Modified bitumen can be produced using additives such as natural rock asphalts, lake asphalts, thermoplastic polymers, latex, and rubber [34]. The bitumen used in this study, produced at the Aliağa Refinery and supplied by the Isparta Municipality asphalt plant, was selected because it represents the commonly used binder in regional road construction projects.

### 2.3. Elvaloy RET

**Figure 2:**
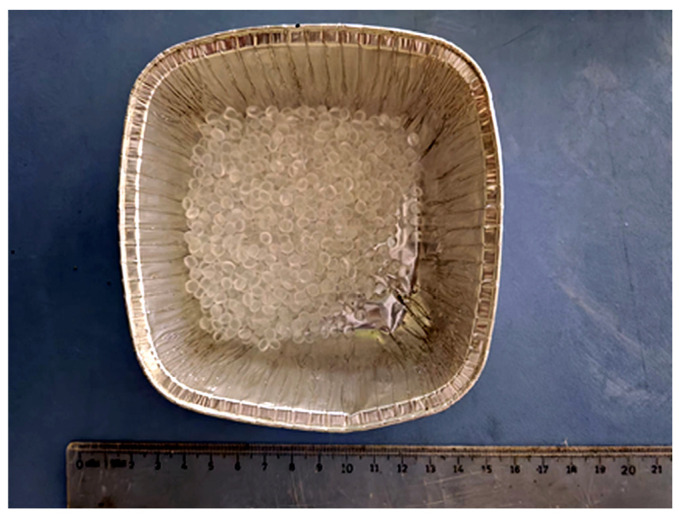
Elvaloy RET used in the study.

Elvaloy RET is an additive used in road pavements and asphalt mixtures to improve the properties of asphalt binders. Elvaloy RET integrates with asphalt binders by providing SHRP-level performance at high temperatures and enhanced flexibility at low temperatures [46]. The general properties of the Elvaloy RET polymer additive used in the modified mixture are presented in Table 3. In this study, Elvaloy RET was tested at 1.6%, 1.7%, 1.8%, and 1.9% modification levels; however, since the mixture with 1.9% did not exhibit homogeneous blending, the study was continued with 1.6%, 1.7%, and 1.8% contents (Figure 2).

### 2.4. Polyphosphoric Acid (PPA)

Polyphosphoric acid (PPA) is an additive used in various industries and plays a significant role in the modification of bituminous binders. It is known that the addition of PPA improves the rheological properties of bitumen, enhances its high-temperature performance, and increases its overall durability. In particular, when used in combination with other modifying additives, PPA improves the workability of bitumen and prevents phase separation during long-term storage [53]. For these reasons, PPA was used in this study at a fixed content of 0.2% in combination with Elvaloy RET for bitumen modification. The properties of the PPA used in the study are presented in Table 4.

### 2.5. Cellulose Fiber

**Figure 3:**
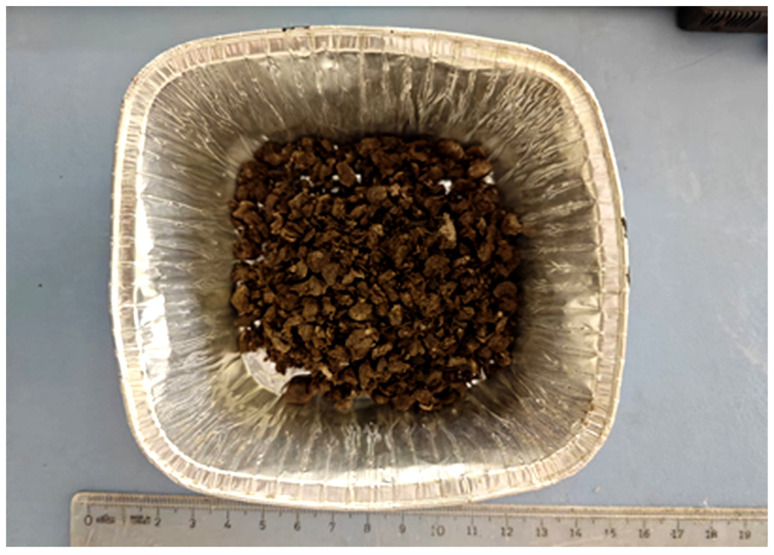
Cellulose fiber used in the study.

Cellulose fibers stand out as one of the most effective stabilizing additives in terms of absorption capacity. Owing to this property, they can strongly retain binders such as bitumen and wax, thereby enhancing the durability of the mixture [54]. The primary reason for preferring cellulose fibers as stabilizers in SMA mixtures is their high absorbent capability. The properties of the cellulose fiber used in this study are presented in Table 5 (Figure 3).

### 2.6. Cellulose Acetate Recycled from Cigarette Butts

**Figure 4:**
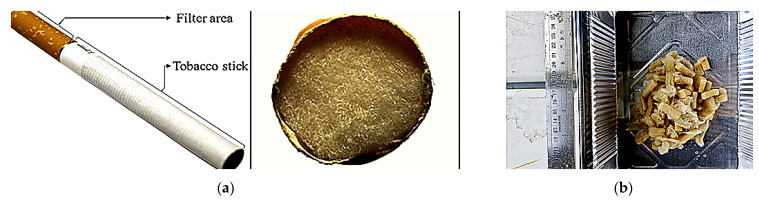
Cross-section of a cigarette filter (**a**) [56], and recycled cellulose acetate used in the study (**b**).

The thermal decomposition behavior of cellulose-based materials follows a similar trend to other biomass materials with high cellulose content. Studies have shown that cigarette filters made of cellulose acetate undergo thermal decomposition within the temperature range of 300–400 °C [55]. In this study, cigarette butts were utilized as a source of recycled cellulose acetate. After being collected from locations such as canteens and cafés, the cigarette butts underwent a cleaning process (discussed in Section 3.1 below) before being incorporated as an additive into the SMA mixtures. The cross-section of a cigarette filter and the recycled cellulose acetate obtained from cigarette butts used in the study are shown in Figure 4.

## 3. Method

### 3.1. Recycling of Waste Cigarette Butts

**Figure 5:**
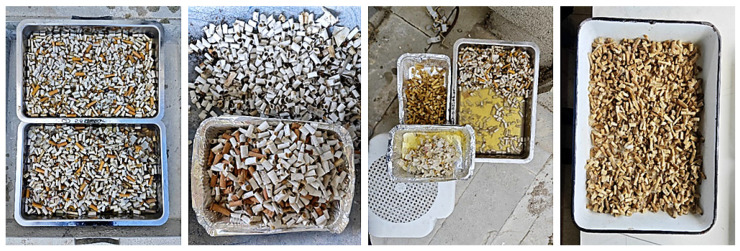
Preliminary cleaning stage of cigarette butts.

Cigarette butts collected from public consumption areas such as canteens, cafés, and restaurants were first immersed in water to remove soluble contaminants from their surfaces and to make them suitable for subsequent chemical treatment processes. The charred portions and tobacco-containing sections were separated from the filter, and the remaining parts were again placed in water to facilitate the easy removal of paper layers (Figure 5).

**Figure 6:**
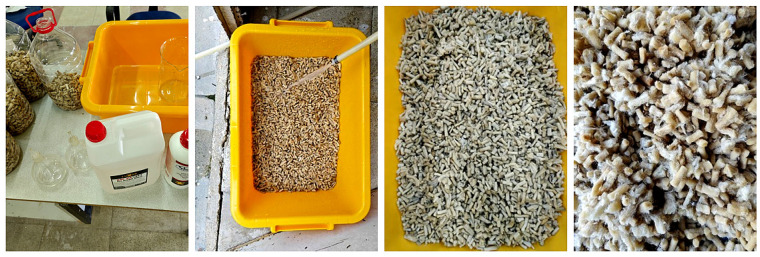
Chemical cleaning process and post-treatment.

During the chemical cleaning stage, a de-esterification mixture consisting of isopropyl alcohol (IPA), perchloroethylene, and 1 mol/L NaOH solution in a 1:1:1 (*v*/*v*) ratio was used. Although this method has not been widely reported in the literature, the use of a similar approach in a previous study [57] supports the applicability of the technique used in this research. In the first step, IPA and perchloroethylene were applied to remove surface contaminants such as nicotine and tar. After the preliminary cleaning, in the second step, the cigarette butts were transferred into the 1:1:1 mixture of IPA, perchloroethylene, and 1 mol/L NaOH solution. The samples were kept in this solution for 2 h, then rinsed thoroughly with water, dried, and stored in airtight containers (Figure 6).

### 3.2. Bitumen Modification

In this study, both pure bitumen and modified bitumen were used. The modification process was carried out using Elvaloy RET at contents of 1.6%, 1.7%, and 1.8%. The bitumen was mixed at 185 °C and a stirring speed of 500 rpm for 2 h. At the end of this period, 0.2% polyphosphoric acid (PPA) was added as a catalyst, and the mixing process was continued for an additional 30 min, completing the modification procedure.

### 3.3. X-Ray Diffraction (XRD) Analysis of Modified Bitumen

X-Ray Diffraction (XRD) analysis is a powerful characterization technique widely used to identify crystalline structures [58]. In addition, it has been extensively applied in the phase analysis of mineral additives used in bitumen and polymer-modified asphalt materials [59,60,61]. To examine the effects of the modifiers used in this study, XRD analysis was performed on bitumen samples modified with 1.7% Elvaloy RET + 0.2% PPA and 1.8% Elvaloy RET + 0.2% PPA.

### 3.4. Bitumen Tests

In this study, both conventional and Superpave bitumen tests were conducted to determine the physical and rheological properties of both pure and modified bitumens. The penetration test was performed to determine the hardness of the bitumen, while the softening point test was carried out to evaluate its flow resistance at high temperatures. In addition, a storage stability test was conducted to evaluate the compatibility between the bitumen and the additives. This method allows the assessment of the uniform dispersion of the additives within the bitumen and the stability of the bitumen during the storage period. The storage stability test of the modified bitumens was carried out in accordance with TS EN 13399 [62]. The bitumen was poured into standard aluminum tubes and kept at 180 °C for 72 ± 1 h, then cooled and divided into three equal parts. The top and bottom sections were used for testing, while the middle part was discarded. According to the HTS [34], the difference in softening point between the top and bottom sections should not exceed 5 °C; otherwise, phase separation is considered to have occurred. In addition, an elastic recovery test was carried out in accordance with TS EN 13398 [63] to determine the ability of the bitumen to return to its original form after deformation. The test is performed at 25 °C using a ductilometer, where the specimen is stretched to 200 mm at a rate of 50 mm/min. The stretched specimen is then cut in half, left to rest for 30 min, and the amount of retraction is measured to calculate the elastic recovery percentage. This method allows for the evaluation of the binder’s flexibility and ability to recover after deformation. To evaluate the high-temperature performance of the binders, the Rolling Thin Film Oven Test (RTFOT) was first conducted to simulate short-term aging, allowing for the analysis of binder stability and workability. Following RTFOT aging, and subsequently after the Pressure Aging Vessel (PAV) procedure, representing long-term aging, the Dynamic Shear Rheometer (DSR) test was performed to determine the rheological performance of the binders. Using the complex shear modulus (G*) and phase angle (δ) values obtained from the DSR, the deformation resistance and elastic behavior level of the binder were calculated. These parameters are critically important for assessing permanent deformation (rutting) and aging susceptibility, particularly at high service temperatures. In the test, the specimen is placed between two plates, one fixed and the other oscillating. The oscillating plate applies an oscillatory shear force to the specimen, and the rheological properties exhibited by the specimen in response to this force are measured. The Bending Beam Rheometer (BBR) test was conducted to determine the stiffness and creep properties of bituminous binders at low temperatures. This test plays an important role in evaluating the resistance of bitumen to low-temperature cracking. In the test, a bitumen beam specimen of specified dimensions is subjected to a constant load for 4 min, and its behavior is monitored to obtain time–creep stiffness and time–deformation curves. The test is typically performed at sub-zero temperatures to assess the cracking risk of bitumen under cold conditions. According to the test criteria, the creep stiffness (S(t)) should be less than 300 Mpa, and the m-value should be greater than 0.300. Meeting these conditions indicates that the bitumen can perform effectively at low temperatures without a significant risk of cracking [64].

### 3.5. SMA Mixture Tests

SMA specimens were prepared in accordance with the Turkish Highway Technical Specifications (HTS), and the Marshall method was used for mix design [34]. Aggregate samples weighing 1050 g were prepared, and bitumen was added in proportions of 5%, 5.5%, 6%, 6.5%, and 7% by weight. Three specimens were produced for each bitumen content. For each specimen, the practical specific gravity (Dp), air void content (Vh), voids in mineral aggregate (VMA), and voids filled with asphalt (Vfa) values were determined, and the Optimum Bitumen Content (OBC) was calculated. The SMA specimen series and aggregate combinations are presented in Table 6.

For each series given in the table, new specimens were prepared at the OBC and subjected to Indirect Tensile Strength (ITS), Tensile Strength Ratio (TSR), Schellenberg bitumen drainage, sand patch, and Cantabro tests. Through these tests, the mixtures were comprehensively evaluated in terms of their mechanical performance, resistance to moisture damage, bitumen drainage characteristics, surface texture, and resistance to particle loss.

The Marshall stability and flow test, as specified in ASTM D1559 [65], is conducted to determine the resistance of bituminous mixtures to plastic deformation. For the test, the prepared specimens are first measured and weighed, then conditioned in a water bath maintained at 60 ± 1 °C for 30–40 min. After conditioning, the specimens are removed from the bath, centered between the loading heads, and subjected to loading at a rate of 50 ± 2 mm/min. During the test, the stability at the moment of specimen failure and the corresponding flow value are recorded. Stability represents the maximum load the mixture can withstand before failure, indicating its resistance to deformation, while flow refers to the vertical deformation that occurs at the point of maximum load [66].

The ITS test is conducted to indirectly determine the tensile strength of asphalt mixtures by applying compressive loads along the vertical diameter of the specimens using a Marshall loading device. The ITS value is determined by calculating the maximum load the specimen can sustain before failure (Equation (1)). The test is performed in accordance with the ASTM D6931-17 standard [67], using a Marshall testing apparatus at a constant temperature of 25 °C and a loading rate of 50.8 mm/min. This test method is highly important for evaluating the resistance of bituminous mixtures to cracking and assessing their behavior under temperature variations [21].

(1) $$\mathrm{ITS}=\frac{2\mathrm{Pmax}}{\pi \mathrm{td}\;}$$

Pmax represents the maximum applied load (kN), t denotes the specimen thickness (mm), and d refers to the specimen diameter (mm).

Moisture sensitivity is one of the key parameters that directly affects the performance of bituminous mixtures. The presence of water or moisture within the pavement weakens the bond between the bitumen and the aggregate, leading to adhesion loss, cracking, and surface deterioration. The AASHTO T283 standard [68] is used to determine the moisture sensitivity of bituminous mixtures, and according to this standard, the prepared specimens are divided into two groups: unconditioned and conditioned. To simulate real environmental conditions, the conditioned specimens are first subjected to vacuum treatment so that 60–80% of their air voids are filled with water. Then, the specimens are wrapped with stretch film and kept in a freezer at −18 °C for 16 h. After freezing, they are placed in a water bath at 60 °C for 24 h, and subsequently, both conditioned and unconditioned specimens are kept in a water bath at 25 °C for 2 h to equilibrate. After completing these procedures, both the unconditioned (ITSdry) and conditioned (ITSwet) specimens are tested using a Marshall loading device to determine ITS. The TSR of the specimens is then calculated using Equation (2). According to the standard, TSR values must be at least 80%, indicating sufficient resistance to moisture damage. Specimens with TSR values below this threshold are considered to have inadequate resistance to moisture-induced damage [69].

(2) $$\mathrm{TSR}=\frac{\mathrm{ITSwet}}{\mathrm{ITSdry}}\times 100$$

ITSwet indicates the indirect tensile strength of the soaked specimens (kPa), while ITSdry represents the indirect tensile strength of the dry specimens (kPa).

**Figure 7:**
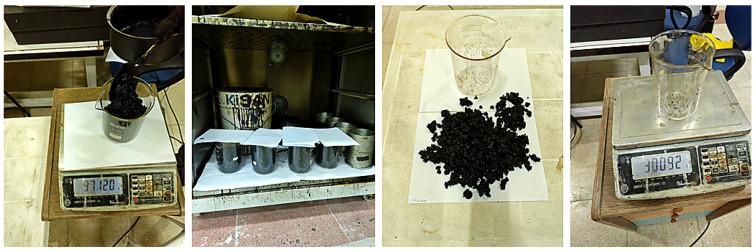
Stages of the bitumen drain-down test.

In the bitumen drain-down test, for conventional bituminous mixtures, 1000 g of SMA mixture prepared at 135 ± 5 °C, and for polymer-modified mixtures (PMB), at 145 ± 5 °C, is placed into a 1000 mL glass beaker that has been preheated to 110 °C. The beaker is weighed with a precision of 0.1 g. After covering the beaker, it is kept in an oven for 1 h ± 1 min at 175 °C for conventional mixtures and 185 °C for modified mixtures. At the end of this period, the beaker is carefully removed from the oven and emptied without shaking. The drained mixture is then weighed again with a precision of 0.1 g, and the weight loss is calculated as a percentage (Figure 7). The test is performed on three parallel mixture samples, and the average of the results is taken for evaluation [34].

**Figure 8:**
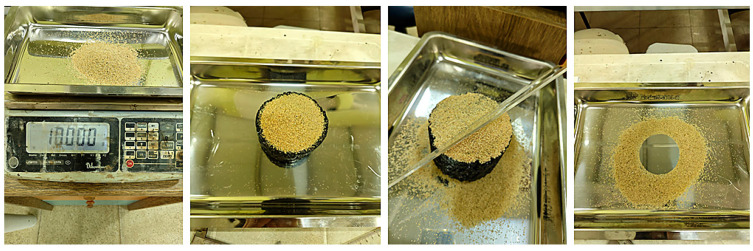
Stages of the sand patch test.

The surface texture roughness of SMA mixtures is determined by measuring the texture depth using the Sand Patch test. In this study, the Sand Patch test was conducted in accordance with the ASTM E965 testing standard [70]. For the test, the average diameter of each SMA briquette was first measured and recorded using a digital caliper. Then, 100 g of sand (with a specific gravity of 1.476 g/cm^3^) was poured onto the surface of the SMA briquette placed on a flat tray. The sand was spread evenly across the surface using a leveling tool to fill the surface voids of the briquette. The weight of the sand filling the surface voids was recorded, and the volume of sand applied to each specimen was calculated (Figure 8). The resulting mean texture depth was determined for each specimen using Equation (3) [53].

According to the HTS, the mean texture depth of the SMA Type-1A wearing course should be greater than 1.0 mm [34].

H = 4V/πD^2^ (3)

H represents the mean texture depth, V refers to the volume of sand used, and D denotes the mean diameter of the sand patch.

**Figure 9:**
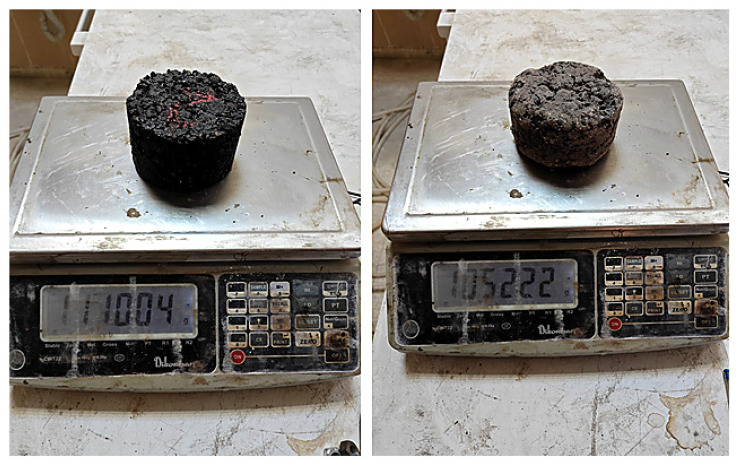
Cantabro test specimen.

In order to evaluate the durability of the mixtures, the Cantabro test was performed. The purpose of this test is to determine the resistance of the mixture to raveling or particle loss. The specimens are placed in the Los Angeles abrasion machine without adding any abrasive load (such as steel balls) and subjected to a total of 300 revolutions (Figure 9). At the end of the test, the initial mass of the specimen is compared with the final mass obtained after the procedure to determine the mass loss [71].

This value is referred to as the Cantabro particle loss (PL) and is calculated using the following equation:

(4) $$\mathrm{PL}\;=\;100\;\times \;\frac{W_{1}-W_{2}}{W_{1}}$$

where W_1_ is the initial mass of the specimen and W_2_ is the final mass after testing.

## 4. Results

### 4.1. X-Ray Diffraction (XRD) Analysis Results of Modified Bitumen

**Figure 10:**
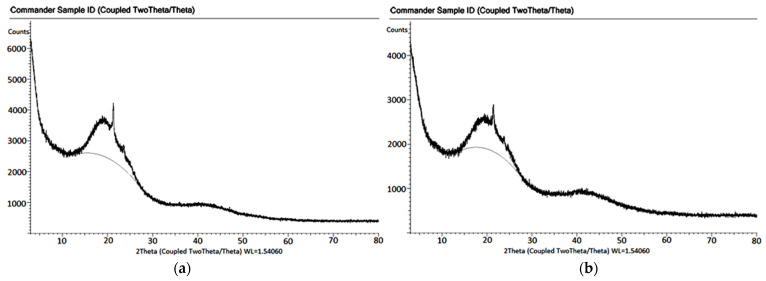
XRD patterns of bitumen modified with 1.7% Elvaloy RET + 0.2% PPA (**a**) and 1.8% Elvaloy RET + 0.2% PPA (**b**).

The X-Ray Diffraction (XRD) analyses of bitumen modified with 1.7% and 1.8% Elvaloy RET + 0.2% PPA are presented together in Figure 10.

In both patterns, a broad peak is observed around 2θ = 20–22°, indicating that the samples largely retain their amorphous character. In both specimens, the intensity decreases significantly beyond 30°, and no strong peak is detected in the 40–80° range. Consequently, it can be stated that neither the E1.7 nor the E1.8 bitumen samples exhibit the formation of a new crystalline phase; however, partial chain rearrangements likely occur, leading to improvements in the viscoelastic and mechanical properties of the material. Furthermore, previous research [10] has shown that Elvaloy RET interacts with the main macromolecular groups within bitumen and exhibits a homogeneous distribution throughout the bitumen phase, which supports the interpretation of the observed structural behavior in this study.

### 4.2. Bitumen Test Results

#### 4.2.1. Conventional Bitumen Test Results

Penetration and softening point tests were performed for pure bitumen and modified bitumens containing different proportions of Elvaloy RET and PPA, and the results are presented in Table 7.

The addition of Elvaloy RET and PPA significantly reduced the penetration value from 58 (for pure bitumen) to approximately 45–48 (a decrease of about 17–22%), indicating a noticeable increase in stiffness. However, further increases in modifier content beyond 1.6% caused only minor variations in penetration, suggesting that the bitumen had reached a relatively stable structure. Similarly, the softening point increased markedly from 51 °C to 60–62.4 °C (an increase of about 18–22%), confirming that Elvaloy RET and PPA substantially enhance the high-temperature stability of the bitumen. Overall, these results demonstrate that the modifiers effectively improve the bitumen’s resistance to deformation and thermal susceptibility.

**Figure 11:**
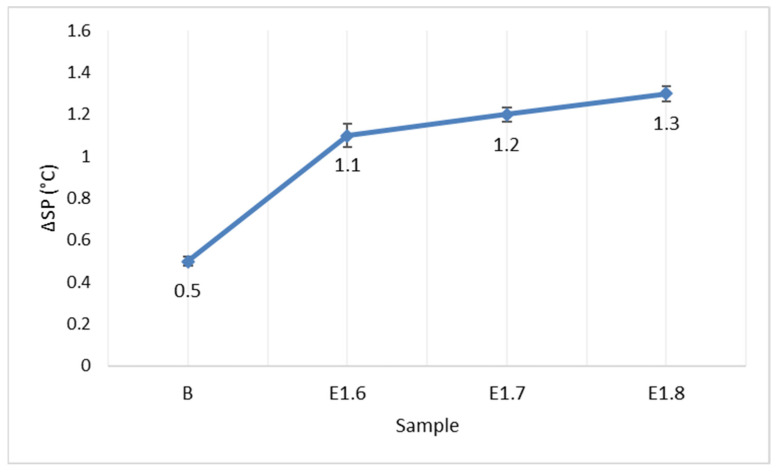
Softening point difference values.

**Figure 12:**
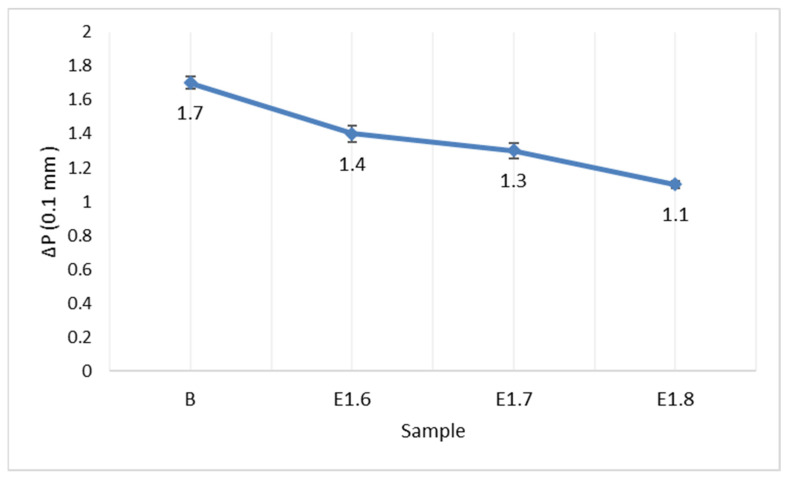
Penetration difference values.

To determine the long-term stability of the interaction between the additives and the bitumen, storage stability tests were conducted. The softening point difference values are presented in Figure 11, and the penetration difference values are shown in Figure 12.

**Figure 13:**
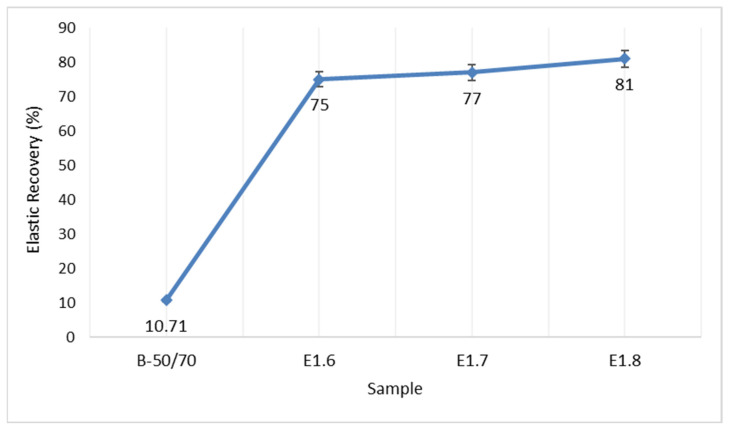
Elastic recovery values.

According to the storage stability test, both pure and Elvaloy RET + PPA-modified bitumens did not undergo phase separation during the storage period. With increasing Elvaloy RET content, the ΔSP and ΔP differences slightly decreased, indicating that the additives mixed compatibly with the bitumen and that the thermal stability of the system improved. The elastic recovery values are presented in Figure 13.

According to the results of the test conducted to determine the elastic recovery properties of bituminous binders, an increase in additive content in the modified bitumen leads to an increase in elastic recovery. This increase indicates that the Elvaloy RET additive promotes the development of an elastic network structure and enhances the material’s ability to return to its original form after deformation.

#### 4.2.2. Results of Mass Loss After RTFOT, Residual Penetration, and Softening Point Tests of Bitumen Samples

To evaluate the short-term aging performance of the bitumens, mass loss, residual penetration, and softening point tests were conducted after the RTFOT procedure (Table 8).

The pure bitumen showed the highest mass loss value of 0.11%, while this value gradually decreased with increasing additive content. In the bitumen modified with 1.8% Elvaloy RET + PPA, the mass loss decreased to 0.02%, indicating the highest short-term aging resistance. As the additive content increased, the penetration value decreased, showing stability in the viscoelastic behavior of the bitumens. The increase in softening point values indicated an improvement in the bitumen’s resistance to heat.

#### 4.2.3. Dynamic Shear Rheometer (DSR) and Bending Beam Rheometer (BBR) Test Results

**Figure 14:**
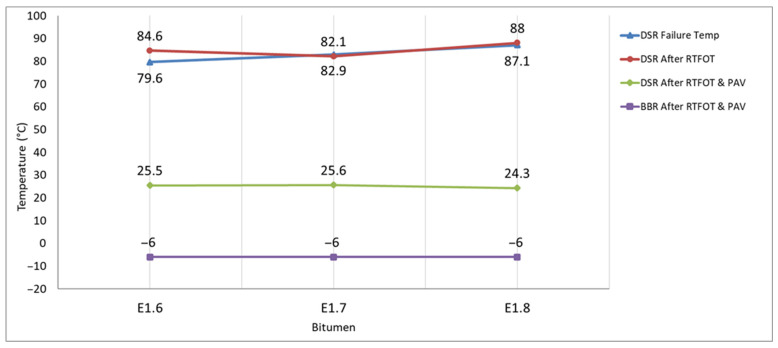
DSR and BBR test results.

The DSR tests were carried out on the bitumens in their unaged state, after short-term aging (RTFOT), and after long-term aging (PAV), and the results are presented in Figure 14.

According to the obtained results, the increase in DSR failure temperatures both before aging and after RTFOT indicates that the bitumens’ deformation resistance at high temperatures has improved, and their resistance to rutting has been strengthened. After PAV aging, the DSR failure temperatures ranged between 24.3 and 25.6 °C, showing that the high-temperature performance was partially preserved and that the additives formed a polymeric network structure, enhancing the rheological strength of the bitumen.

### 4.3. Mixture Test Results

#### 4.3.1. Marshall Stability, Flow and Marshall Quotient Results

In order to determine the Optimum Bitumen Content (OBC), the Marshall Stability (MS) test was performed, and the corresponding values of air voids (Vh), voids filled with bitumen (Vfa), voids in mineral aggregate (VMA), and bulk specific gravity (Dp) were obtained for each specimen. Each data point represents the average of three replicate measurements. Standard deviations were calculated and found to be small, indicating high repeatability of the results. Therefore, only mean values are displayed in the figures to enhance visual clarity. In SMA mixtures, the optimum bitumen content is generally determined based on the Vh value. In the specification, the air void limits for hot climate regions are in the range of 3–4% [34]. A target value of 3.5% was adopted, and the optimum bitumen content for each series was determined based on the condition of Vh = 3.5%. Therefore, the air void content at the optimum bitumen content is 3.5% for all series. Detailed air void data corresponding to each bitumen content level (5.0%, 5.5%, 6.0%, 6.5%, and 7.0%) used in determining the optimum binder content are presented in Table 9.

**Figure 15:**
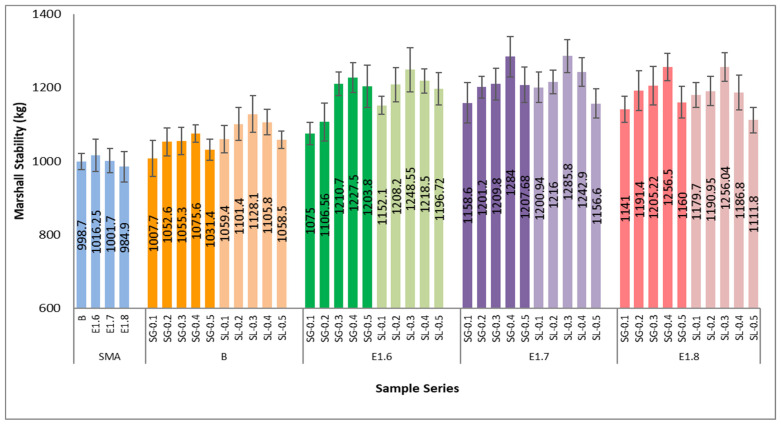
Marshall stability values of all SMA series.

**Figure 16:**
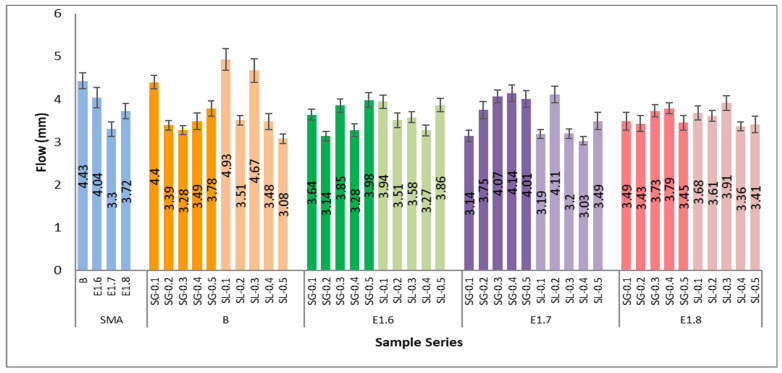
Flow values of all SMA series.

When all series are generally examined, it is observed that the Vh values decrease as the bitumen content increases. This trend indicates that the increasing amount of bitumen in the mixture fills the voids between the aggregates, resulting in a more compact structure. In mixtures with pure bitumen (B series), Vh values ranged between 4.61 and 6.90% at 5% bitumen content and decreased to 0.77–1.73% at 7% bitumen content. In the E1.6 series, the void ratios ranged from 4.83 to 7.82% at 5% bitumen content and declined to 1.00–3.71% at 7%. For the E1.7 series, these values were recorded as 5.09–7.71% at 5% bitumen content and 1.32–3.80% at 7%, while in the E1.8 series, they started from 5.06 to 7.40% and decreased to 1.08–3.77% at 7%. The SG fiber-modified mixtures generally exhibited higher void ratios than the SL fiber-modified ones due to the porous nature of the SG fibers. On the other hand, SL fiber-modified mixtures provided better interaction with bitumen, resulting in lower Vh values and a denser, more durable structure. In the SMA series (B-SMA, E1.6-SMA, E1.7-SMA, E1.8-SMA), void ratios ranged from 3.89 to 4.91% at 5% bitumen content and decreased to 0.36–1.30% at 7%. In Figure 15, the Marshall stability values at the optimum bitumen content for each series are presented, while Figure 16 shows the corresponding flow values.

According to the Marshall test results, a significant increase in the Marshall stability values of the mixtures was observed with bitumen modification and fiber additives. In the SMA (reference) series, the average stability value was approximately 1000 kg, while it increased by about 18–19% to 1185 kg in the E1.6 series, by approximately 22% to 1216 kg in the E1.7 series, and by around 19% to 1188 kg in the E1.8 series. These results indicate that the use of Elvaloy RET, PPA, and SG-SL additives significantly enhances the load-bearing capacity of the mixtures. In particular, the E1.7 series achieved the optimum balance between high stability and workability, exhibiting the best overall performance among the fiber-modified mixtures.

**Figure 17:**
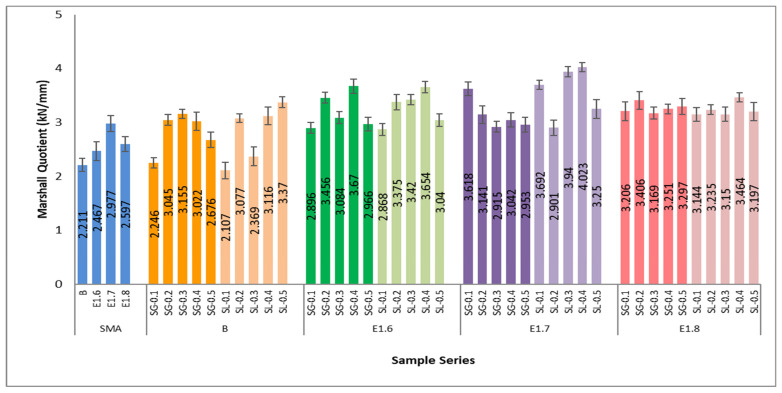
Marshall quotient values of all SMA series.

As the modification ratio increases, a decrease in flow values is observed. In the SMA (pure bitumen) mixtures, the average flow ranged between 4.0 and 4.4 mm, while in the E1.6 series, it decreased to 3.1–3.9 mm; in the E1.7 series, to 3.1–4.1 mm; and in the E1.8 series, to around 3.3–3.8 mm. This indicates a 15–25% reduction in deformation due to bitumen modification. Mixtures containing SG additive exhibited lower flow values, indicating higher stiffness and deformation resistance compared to SL fiber-modified mixtures. In terms of modification level, the E1.7 modification provided the most suitable balance for achieving optimum performance. In Figure 17, the Marshall quotient (MQ) values obtained from the Marshall stability and flow results of the SMA series are presented.

In the SMA series, the average Marshall quotient value ranged between 2.2 and 2.6 kN/mm, while in the E1.6–E1.8 series, these values increased to around 3.2–3.3 kN/mm, corresponding to an improvement of approximately 45–50% compared to the reference mixture. This increase indicates an improvement in both the load-bearing capacity and the resistance to permanent deformation of the mixtures. The rise in the Marshall quotient is directly associated with enhanced rutting resistance, as widely reported in the literature. It is observed that mixtures containing SG and SL additives exhibit a lower potential for permanent deformation compared to the reference mixtures prepared with pure bitumen. In particular, the E1.7 series achieved the optimum Marshall quotient, combining high stability with balanced flow values.

#### 4.3.2. Indirect Tensile Strength and Moisture Damage Resistance Test Results

**Figure 18:**
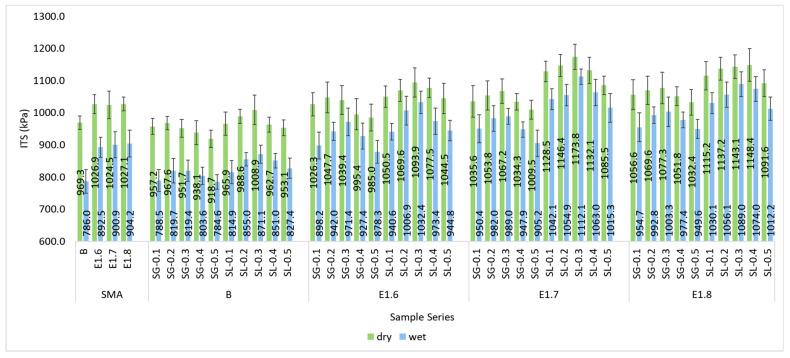
ITS values of all mixture series.

**Figure 19:**
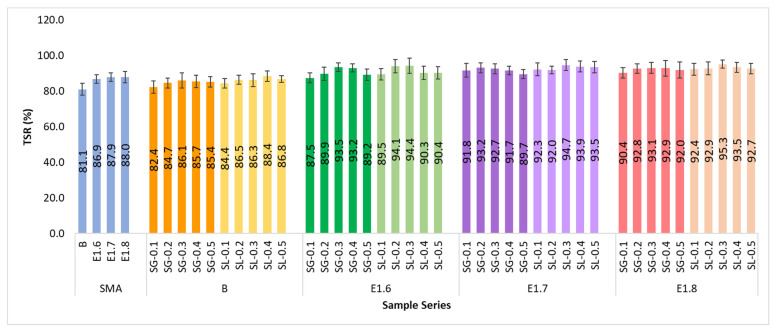
TSR values of all mixture series.

In the study, ITS tests were performed on both conditioned and unconditioned specimens to determine their resistance to moisture-induced pavement damage. ITS values of all series are presented in Figure 18, while the corresponding TSR values are shown in Figure 19.

A meaningful improvement in ITS values is observed with the increase in the modification rate. In the SMA series, the average dry ITS value was approximately 1012 kPa, while in the E1.6, E1.7, and E1.8 series, these values increased to 1043, 1087, and 1092 kPa, respectively. Similarly, the wet ITS values ranged between 870 and 1014 kPa, indicating that the mixtures retained about 91–93% of their strength. This enhancement can be attributed to the micro-reinforcement effect of fibers and the improved adhesion between bitumen and aggregate. The small difference between the dry and wet specimen results indicates that the modification reduced the moisture sensitivity of the mixtures, suggesting that they will be more resistant to moisture-induced stripping, rutting, and other forms of deterioration throughout their service life. The TSR values of the series are presented in Figure 19.

As the modification ratio increases, TSR values rise, indicating a reduction in moisture sensitivity. In the SMA series, the TSR values ranged between 81 and 88%, while in the modified mixtures, these values exceeded 90%, reaching approximately 93% in the E1.7 and E1.8 series. This increase corresponds to an improvement of about 12–14%. This result demonstrates that the modification and fiber additives impart a hydrophobic character to the mixtures, preventing water from weakening the binder. Among the modified mixtures, SG-added mixtures exhibited higher stiffness and durability, whereas SL-added mixtures showed the best performance in terms of moisture resistance. In particular, the E1.7 series achieved the optimum performance level.

A paired *t*-test was applied to examine the difference between dry and wet samples, a one-way ANOVA with Tukey’s test was used to compare modification levels, an independent *t*-test was conducted to evaluate the difference between fiber types, and a regression analysis was performed to determine the relationship between fiber content and TSR.

According to the analysis results, moisture conditioning caused an average decrease of approximately 10% (about 101 kPa) in ITS values, and this reduction was found to be statistically significant (*p* < 0.001). The Elvaloy RET + PPA modification significantly increased TSR values, raising the average TSR from 86% in the control mixtures to about 93% in the modified mixtures (*p* < 0.001). No statistically significant difference was found between SG and SL fibers in terms of TSR (difference < 2%, *p* > 0.05). Although the effect of fiber content (0.1–0.5%) on TSR was not linearly significant, the highest values were obtained at approximately 0.3–0.4%. According to the regression analysis results, a slight increase in TSR was observed with increasing fiber content for both fiber types. For the SG fiber, the relationship was defined by the equation TSR = 85.42 + 16.23·(Fiber%), with R^2^ = 0.78 (*p* = 0.041). For the SL fiber, the relationship was determined as TSR = 86.01 + 13.55·(Fiber%), with R^2^ = 0.72 (*p* = 0.056). Although the increase was not completely linear, the highest TSR values were observed at a fiber content of 0.3–0.4%, indicating the occurrence of a saturation effect beyond this level. In conclusion, SMA mixtures modified with Elvaloy RET + PPA exhibited high resistance to moisture-induced damage, and the SG fiber performed as effectively as the SL fiber.

#### 4.3.3. Bitumen Drain-Down Test Results

**Figure 20:**
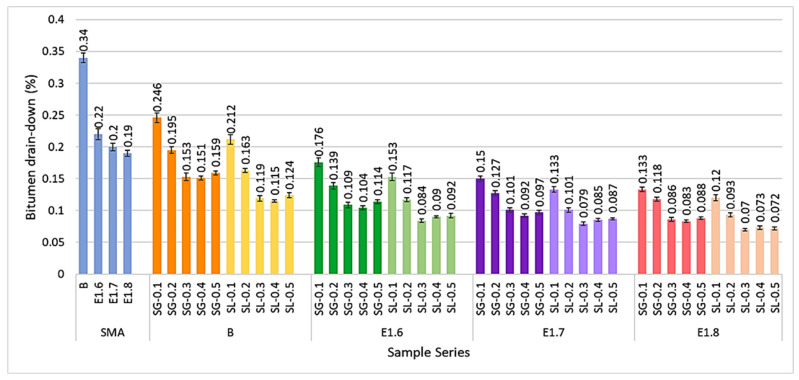
Bitumen drain-down percentage values for the mixture series.

Figure 20 presents the bitumen drain-down percentages for all mixture series.

In the test conducted to determine the amount of bitumen drainage from the mineral aggregate, it was generally observed that both SG and SL additives significantly reduced the bitumen drainage rate. While the SMA mixtures exhibited high drainage values of around 0.34%, the modified mixtures reduced this rate to below 0.1%. This difference can be attributed to the increased viscosity of the modified binder and the micro-porous structure of the fibers, which physically retain the bitumen. Furthermore, the bitumen drainage values of SL-modified mixtures were lower compared to those of the SG series. Due to their larger surface area, SL fibers effectively absorbed the bitumen phase and minimized drainage.

#### 4.3.4. Sand Patch Test Results

**Figure 21:**
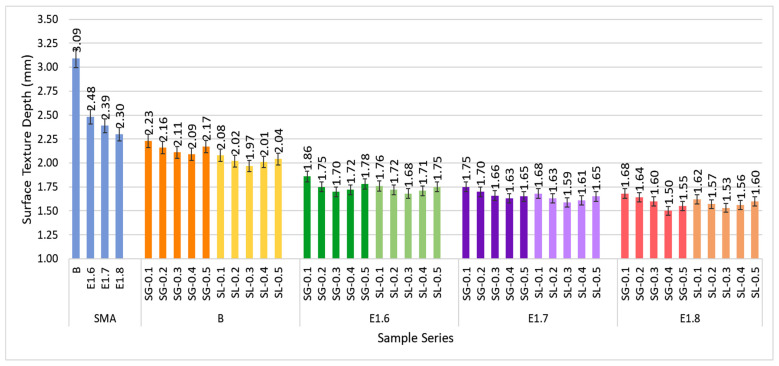
Sand Patch test results of all SMA series.

In the test conducted to determine the surface texture roughness of the SMA mixtures, the surface texture depth values of all series are presented in Figure 21.

In the graph, it is observed that the surface texture depth (STD) values decrease with increasing fiber content and modification ratio. In the B-SMA series, the surface texture depth was 3.09 mm, and as the modification rate increased, this value gradually decreased to 2.30 mm in the E1.8 series, corresponding to a 26% reduction. In the fiber-modified mixtures, both the SG and SL series exhibited lower surface roughness, with average values ranging between 1.6 and 1.8 mm. This represents a reduction of approximately 40–50% compared to the unmodified SMA mixture. This decrease in STD indicates that the voids between aggregates are reduced and the bitumen is distributed more uniformly on the surface. As the modification ratio increases, a reduction in STD values is observed for both fiber types, which can be explained by the densification of the mixture. The STD values of the SL fiber-modified mixtures are slightly lower compared to those of the SG series. This can be attributed to the more elastic structure of cellulose fibers, which creates a smoother surface texture within the mixture.

#### 4.3.5. Cantabro Test Results

**Figure 22:**
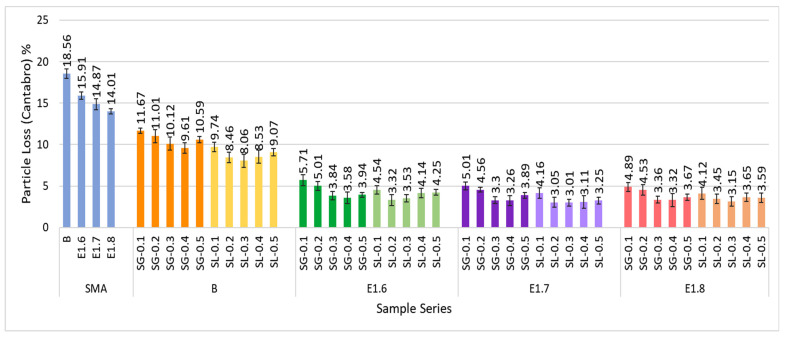
Cantabro test results of all SMA series.

The test results obtained to measure the resistance of the SMA mixtures against fragmentation are presented in Figure 22.

When the mixtures are evaluated as a whole, it is observed that the amount of particle loss decreases with the increase in the modification ratio. This improvement can be attributed to the enhanced viscosity of the modified bitumen and the better coating of aggregates due to the reinforcing effect of the fibers. The SL fiber-modified mixtures exhibited slightly lower particle loss compared to the SG fiber-modified mixtures, which can be explained by the micro-bonding reinforcement effect of cellulose fibers that enhances the cohesion within the mixture. Both types of additives provided more than a 70% improvement in abrasion resistance compared to the mixtures prepared with pure bitumen.

#### 4.3.6. Cost Analysis

In this study, the cost analysis was conducted based solely on the bitumen, Elvaloy RET, and fiber components (either industrial cellulose or cellulose acetate fibers recovered from cigarette butts) contained in the mixtures. The unit prices were taken as follows: bitumen $0.37/kg, Elvaloy RET $6.01/kg, industrial cellulose fiber $0.60/kg, and SG fiber recovered from cigarette waste $0.29/kg. The SG fibers are produced through a chemical cleaning and recovery process; therefore, their pricing was determined based on the cleaning and processing costs. The cost analysis was performed by comparing each SMA series containing SG fibers with the corresponding SMA mixture containing the same amount of SL, which was taken as the reference. For each mixture ratio within the series, the optimum bitumen percentage, bitumen quantity (kg), bitumen cost, Elvaloy RET cost, fiber cost, and total cost were calculated. The average values of the five mixture ratios were then summarized and presented in the corresponding columns of the cost comparison tables for each series (Table 10).

In all series, the SG fiber-modified mixtures were found to be, on average, 1.7–2.5 $/t less costly than the corresponding SL fiber-modified mixtures. The largest cost difference was observed in the E1.8 series (≈2.5 $/t), while the smallest difference occurred in the E1.7 series (≈1.6 $/t). Overall, the use of SG fibers in SMA mixtures provided a cost saving of approximately 6–8% compared to the use of conventional SL fibers.

## 5. Conclusions

In this study, the usability of cellulose acetate (SG) fibers recycled from cigarette butts as an alternative to the conventional cellulose (SL) fibers commonly used in the literature was investigated in Stone Mastic Asphalt (SMA) mixtures. In addition to the binder tests applied to neat and Elvaloy RET + PPA-modified bitumens, the performance tests carried out on SMA mixtures were also comprehensively evaluated, and significant improvements were obtained at both levels.

In terms of mixture performance, the combined use of fibers and modified bitumen provided notable improvements in Marshall stability, Marshall quotient, indirect tensile strength (ITS) and tensile strength ratio (TSR) values. Compared to SMA mixtures prepared with neat bitumen, stability performance increased significantly in the series where fibers and modified bitumen were used. Regarding ITS, the best results were obtained in the E1.6 series in mixtures where both SG and SL fibers were used; TSR values reached the highest levels, especially in the E1.6 and E1.7 mixtures.

Binder drainage decreased to the lowest levels in mixtures where fibers and modification were used together, and both fiber types effectively retained the bitumen, significantly reducing binder loss. Surface texture and Cantabro particle loss results showed that the fibers exhibited a homogeneous distribution within the mixture, reduced surface roughness, and significantly improved the mixture’s particle loss resistance when used at certain proportions. The cost analysis revealed that SG fibers offer a generally more economical option compared to traditional SL fibers in terms of production and application.

As a result, recycled cellulose acetate fibers stand out as an additive that, when used at low dosages (0.3–0.4%), improves bitumen retention, reduces drainage, enhances the mechanical performance of the mixture, and provides cost advantages without adversely affecting the workability of SMA mixtures. However, for widespread industrial-scale use, the production process needs to be optimized, fiber properties need to be standardized, and the environmental impacts of the additive should be investigated within the scope of life-cycle assessment. In addition, considering the lack of literature on the recycling performance of SMA mixtures containing SG fibers, it is recommended that this issue be supported with more comprehensive studies in the future.

## Figures and Tables

**Table 1:** Physical properties of aggregates.

Test	Result		Standard
Coarse Aggregate Apparent Specific Gravity (g/cm^3^)	2.680		TS EN 1097-6 [35]
Coarse Aggregate Bulk Specific Gravity (g/cm^3^)	2.667		TS EN 1097-6 [35]
Coarse Aggregate Water Absorption (%)	0.4%		TS EN 1097-6 [35]
Fine Aggregate Apparent Specific Gravity (g/cm^3^)	2.662		TS EN 1097-6 [35]
Fine Aggregate Bulk Specific Gravity (g/cm^3^)	2.614		TS EN 1097-6 [35]
Fine Aggregate Water Absorption (%)	0.6%		TS EN 1097-6 [35]
Filler Specific Gravity (g/cm^3^)	2.610		TS EN 1097-7 [36]
Effective Specific Gravity of Mixture (Experimental)	2.666		ASTM D-2041 [37]
Effective Specific Gravity of Mixture (Calculated)	2.658		-
**Test**	**Result**	**HTS Requirement (SMA Wearing Course)** [34]	**Standard**
Resistance to Fragmentation (Los Angeles Abrasion Loss, %)	20	≤25	AASHTO T-96 [38]
Abrasion Resistance (Micro-Deval, %)	9.1		TS EN 1097-1 [39]
Durability Against Weathering (MgSO_4_, Freeze–Thaw Loss, %)	1.6	≤14	TS EN 1367-2 [40]
Flakiness Index (%)	17	≤25	BS 812 [41]
Stripping Resistance (%)	70–75	≥60	ASTM D1664 [42]
Methylene Blue Value (g/kg, of Mixture)	1.5	≤1.5	TS EN 933-9 [43]

**Table 2:** Aggregate gradation of SMA mixture.

Sieve Diameter		SMA Type-1A Mixture Gradation [34]		Sample Weight: 1050 g	
Inch	mm	% Passing	% Retained	Retained Weight (g)	Cumulative Weight (g)
¾”	19.0	100.0	-	-	-
½”	12.5	93.5	6.5	68.25	68.25
3/8”	9.5	69.9	23.6	247.8	316.05
No. 4	4.75	34.1	35.8	375.9	691.95
No. 10	2.00	23.4	10.7	112.35	804.30
No. 40	0.425	15.4	8	84	888.30
No. 80	0.180	11.9	3.5	36.75	925.05
No. 200	0.075	9.2	2.7	28.35	953.40
Pan (Below 0.075 mm)	-	9.2	96.6	1050	

**Table 3:** General properties of Elvaloy RET polymer [46].

Physical Properties		
Property	Nominal Values	Test Method(s)
Density (ρ)	0.95 g/cm^3^	ASTM D792/ISO 1183 [47,48]
Melt Flow Index (190 °C/2.16 kg)	12 g/10 min	ASTM D1238/ISO 1133 [49,50]
Thermal Properties		
Property	Nominal Values	Test Method(s)
Melting Point (DSC)	80 °C (176 °F)	ASTM D3418/ISO 3146 [51,52]
Freezing Point (DSC)	55 °C (131 °F)	ASTM D3418/ISO 3146 [51,52]
Processing Information		
Property	Value	
Maximum Processing Temperature	220 °C (428 °F)	

**Table 4:** Properties of PPA [53].

Property	Value
Chemical Formula	H_n+2_P_n_O_3n+1_
Physical Form	Liquid
Concentration	% ≥ 90– ≤ 100
Metal Corrosion Rate	May corrode metals

**Table 5:** Technical properties of cellulose fiber [53].

Appearance	Thickness: 4–7 mm
Schellenberg Drain-down Value	Maximum 0.18% (Specification Limit: 0.3%)
Moisture Content	Maximum 5%
Oil Absorption Capacity	At least 5 times the weight of cellulose

**Table 6:** SMA specimen series and aggregate combinations.

Sample Code	Description	Sample Code	Description
E1.6-SG-0.1	%1.6 Elvaloy + %0.2 PPA + SG %0.1	E1.6-SL-0.1	%1.6 Elvaloy + %0.2 PPA + SL %0.1
E1.6-SG-0.2	%1.6 Elvaloy + %0.2 PPA + SG %0.2	E1.6-SL-0.2	%1.6 Elvaloy + %0.2 PPA + SL %0.2
E1.6-SG-0.3	%1.6 Elvaloy + %0.2 PPA + SG %0.3	E1.6-SL-0.3	%1.6 Elvaloy + %0.2 PPA + SL %0.3
E1.6-SG-0.4	%1.6 Elvaloy + %0.2 PPA + SG %0.4	E1.6-SL-0.4	%1.6 Elvaloy + %0.2 PPA + SL %0.4
E1.6-SG-0.5	%1.6 Elvaloy + %0.2 PPA + SG %0.5	E1.6-SL-0.5	%1.6 Elvaloy + %0.2 PPA + SL %0.5
E1.7-SG-0.1	%1.7 Elvaloy + %0.2 PPA + SG %0.1	E1.7-SL-0.1	%1.7 Elvaloy + %0.2 PPA + SL %0.1
E1.7-SG-0.2	%1.7 Elvaloy + %0.2 PPA + SG %0.2	E1.7-SL-0.2	%1.7 Elvaloy + %0.2 PPA + SL %0.2
E1.7-SG-0.3	%1.7 Elvaloy + %0.2 PPA + SG %0.3	E1.7-SL-0.3	%1.7 Elvaloy + %0.2 PPA + SL %0.3
E1.7-SG-0.4	%1.7 Elvaloy + %0.2 PPA + SG %0.4	E1.7-SL-0.4	%1.7 Elvaloy + %0.2 PPA + SL %0.4
E1.7-SG-0.5	%1.7 Elvaloy + %0.2 PPA + SG %0.5	E1.7-SL-0.5	%1.7 Elvaloy + %0.2 PPA + SL %0.5
E1.8-SG-0.1	%1.8 Elvaloy + %0.2 PPA + SG %0.1	E1.8-SL-0.1	%1.8 Elvaloy + %0.2 PPA + SL %0.1
E1.8-SG-0.2	%1.8 Elvaloy + %0.2 PPA + SG %0.2	E1.8-SL-0.2	%1.8 Elvaloy + %0.2 PPA + SL %0.2
E1.8-SG-0.3	%1.8 Elvaloy + %0.2 PPA + SG %0.3	E1.8-SL-0.3	%1.8 Elvaloy + %0.2 PPA + SL %0.3
E1.8-SG-0.4	%1.8 Elvaloy + %0.2 PPA + SG %0.4	E1.8-SL-0.4	%1.8 Elvaloy + %0.2 PPA + SL %0.4
E1.8-SG-0.5	%1.8 Elvaloy + %0.2 PPA + SG %0.5	E1.8-SL-0.5	%1.8 Elvaloy + %0.2 PPA + SL %0.5
**Reference Samples**			
B-SG-0.1	Pure bitumen + SG %0.1	B-SL-0.1	Pure bitumen + SL %0.1
B-SG-0.2	Pure bitumen + SG %0.2	B-SL-0.2	Pure bitumen + SL %0.2
B-SG-0.3	Pure bitumen + SG %0.3	B-SL-0.3	Pure bitumen+ SL %0.3
B-SG-0.4	Pure bitumen + SG %0.4	B-SL-0.4	Pure bitumen + SL %0.4
B-SG-0.5	Pure bitumen + SG %0.5	B-SL-0.5	Pure bitumen + SL %0.5
B-SMA		Pure bitumen (unmodified)-SMA	
E1.6-SMA		%1.6 Elvaloy + %0.2 PPA (unmodified)-SMA	
E1.7-SMA		%1.7 Elvaloy + %0.2 PPA (unmodified)-SMA	
E1.8-SMA		%1.8 Elvaloy + %0.2 PPA (unmodified)-SMA	

**Table 7:** Penetration and softening point values.

Sample	Elvaloy RET (%)	PPA (%)	Penetration (0.1 mm)	Softening Point (°C)
B	0.0	0.0	58	51.0
E1.6	1.6	0.2	45	60.0
E1.7	1.7	0.2	47	61.0
E1.8	1.8	0.2	48	62.4

**Table 8:** Test results of bitumen samples after RTFOT.

Sample Code	Mass Loss (%)	Residual Penetration (%)	Softening Point (°C)
B-50/70	0.11	81.2	53.8
E1.6	0.05	75.6	63.0
E1.7	0.03	70.2	64.0
E1.8	0.02	64.0	66.4

**Table 9:** Relationship between bitumen content and Vh.

Bitumen Content	Vh (%)										
	E1.6-SG-0.1	E1.6-SG-0.2	E1.6-SG-0.3	E1.6-SG-0.4	E1.6-SG-0.5	E1.6-SL-0.1	E1.6-SL-0.2	E1.6-SL-0.3	E1.6-SL-0.4	E1.6-SL-0.5
5%	4.84	5.05	6.07	6.42	6.68	4.83	4.97	7.03	7.82	7.78
5.5%	4.09	3.93	4.58	5.39	5.42	3.09	3.35	5.57	5.32	5.69
6%	2.71	3.01	2.97	4.28	3.25	1.53	1.50	4.30	4.24	4.19
6.5%	1.36	1.69	1.52	3.38	3.55	1.30	1.30	3.15	3.29	3.26
7%	1.11	1.32	1.23	3.04	3.71	1.00	1.00	2.74	2.76	2.92
	E1.7-SG-0.1	E1.7-SG-0.2	E1.7-SG-0.3	E1.7-SG-0.4	E1.7-SG-0.5	E1.7-SL-0.1	E1.7-SL-0.2	E1.7-SL-0.3	E1.7-SL-0.4	E1.7-SL-0.5
5%	5.09	5.99	7.51	7.71	6.78	5.89	5.93	7.30	7.47	7.55
5.5%	3.81	4.58	5.47	6.24	5.51	4.82	4.90	5.47	5.51	5.59
6%	2.97	3.16	3.71	5.02	3.34	3.71	3.79	4.12	3.99	4.08
6.5%	1.61	2.15	2.59	3.01	3.66	2.68	2.72	2.72	3.09	3.17
7%	1.32	1.85	2.35	3.67	3.80	1.89	1.94	2.43	1.40	1.48
	E1.8-SG-0.1	E1.8-SG-0.2	E1.8-SG-0.3	E1.8-SG-0.4	E1.8-SG-0.5	E1.8-SL-0.1	E1.8-SL-0.2	E1.8-SL-0.3	E1.8-SL-0.4	E1.8-SL-0.5
5%	5.06	5.58	6.88	7.19	6.89	6.21	6.29	7.40	7.27	7.35
5.5%	3.80	4.16	4.85	5.64	4.97	5.32	5.34	5.43	5.68	5.77
6%	2.71	3.01	3.31	4.45	3.81	4.39	4.43	3.91	4.13	4.22
6.5%	1.81	2.12	2.26	3.63	3.41	3.42	3.57	2.85	2.63	2.71
7%	1.08	1.50	1.69	3.17	3.77	2.43	2.77	2.26	1.16	1.24
	B-SG-0.1	B-SG-0.2	B-SG-0.3	B-SG-0.4	B-SG-0.5	B-SL-0.1	B-SL-0.2	B-SL-0.3	B-SL-0.4	B-SL-0.5
5%	4.61	4.72	4.60	5.17	5.01	4.62	6.52	6.74	5.29	6.90
5.5%	2.67	3.28	3.56	3.81	3.89	3.60	3.88	4.62	3.81	3.64
6%	2.03	2.15	2.40	2.60	2.97	2.38	2.78	2.69	2.40	2.52
6.5%	1.71	1.61	1.36	1.81	1.65	1.40	2.03	1.60	1.73	1.15
7%	0.99	1.23	1.07	1.27	1.27	1.02	0.85	0.77	1.40	1.73
				B-SMA	E1.6-SMA	E1.7-SMA	E1.8-SMA			
5%				4.04	3.89	4.60	4.91			
5.5%				2.87	3.14	3.56	3.49			
6%				1.87	2.46	2.40	2.35			
6.5%				0.85	1.73	1.36	1.48			
7%				0.36	1.30	1.07	0.89			

**Table 10:** Average cost analysis of all SMA series.

Series	Opt. Bitumen (%)	Bitumen (kg)	Bitumen Cost ($)	Elvaloy Cost ($)	Fiber Cost ($)	Total ($/t)	Difference in SG Compared to SL ($)
B-SG	5.48	54.76	20.37	0	0.87	21.24	−1.68 (-cost saving)
B-SL	5.62	56.56	20.89	0	1.8	22.92	
E1.6-SG	6.02	59.46	22.16	5.73	0.87	28.76	−2.15 (-cost saving)
E1.6-SL	6.14	61.42	22.87	5.91	1.8	30.91	
E1.7-SG	6.08	60.82	22.63	6.21	0.87	29.8	−1.64 (-cost saving)
E1.7-SL	6.17	61.64	22.96	6.31	1.8	31.44	
E1.8-SG	6.04	60.78	22.46	6.65	0.87	30	−2.53 (-cost saving)
E1.8-SL	6.33	63.32	23.59	6.85	1.8	32.53	

## Data Availability

The original contributions presented in this study are included in the article. Further inquiries can be directed to the corresponding author.
